# Systematic and efficient navigating potential energy surface: Data for silver doped gold clusters

**DOI:** 10.1016/j.dib.2016.04.014

**Published:** 2016-04-11

**Authors:** Vitaly V. Chaban

**Affiliations:** Federal University of Sao Paulo, Brazil

**Keywords:** Global minimum, Gold, Nanocluster, Simulation

## Abstract

Locating global minimum of certain atomistic ensemble is known to be a highly challenging and resource consuming task. This dataset represents joint usage of the semi-empirical PM7 Hamiltonian, Broyden–Fletcher–Goldfarb–Shanno algorithm and basin hopping scheme to navigate a potential energy surface. The Au_20_ nanocluster was used for calibration as its global minimum structure is well-known. Furthermore, Au_18_Ag_2_ and Au_15_Ag_5_ were simulated for illustration of the algorithm performance. The work shows encouraging results and, particularly, underlines proper accuracy of PM7 as applied to this type of heavy metal systems. The reported dataset motivates to use the benchmarked method for studying potential energy surfaces of manifold systems and locate their global-minimum atomistic configurations.

## Specification Table

**Table:** 

Subject area	*Physics*, *Chemistry*
More specific subject area	*Computational Molecular Science*
Type of data	*Tables, figures*
How data was acquired	*Computer simulation*
Data format	*Filtered*, *analyzed*
Experimental factors	*Computer simulations at the Neumann supercomputing cluster*
Experimental features	*Computer simulations at the Neumann supercomputing cluster*
Data source location	*São Paulo, Brazil*
Data accessibility	*All data are in this article*

## Value of the data

• Since accuracy of PM7 was proven for gold nanoclusters, this method can be applied to other important problems in materials chemistry.
• Knowledge regarding low-energy local minima of gold nanoclusters is useful to develop future synthetic methods and identify experimental structures.
• The reported geometries and formation energies for unusual conformers can be elaborated using higher-level methods in computational chemistry.

## 1. Data

The paper reports possible local-minima structures of Au_20_, Au_18_Ag_2_, Au_15_Ag_5_ obtained from the potential energy surface scans. Starting from an arbitrary geometry, a correct structure of the Au_20_ nanocluster will be found (an experimental global-minimum conformation of Au_20_ is known to be a pyramid with the *T_d_* point group symmetry) [1].

## 2 Experimental design, materials and methods

**Fig. 1:**
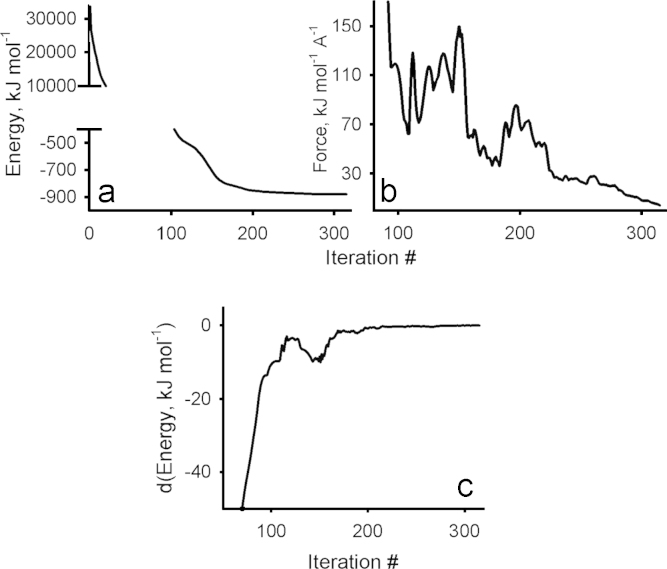
Geometry optimization of the silver doped gold nanoclusters. The minimized formation heat corresponds to local minimum structures.

**Fig. 2:**
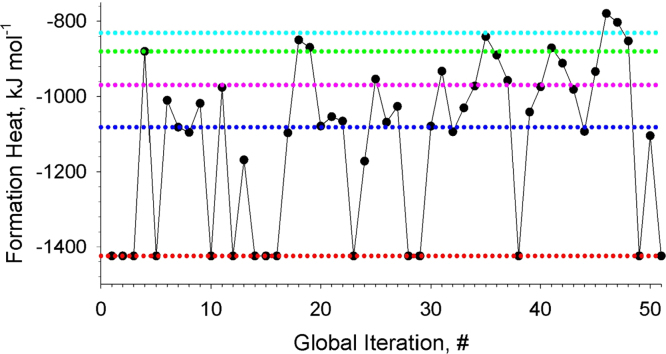
Formation energies of the revealed local-minimum structures of Au_20_. The dotted color lines depict energies of the structures, which were obtained most frequently.

**Fig. 3:**
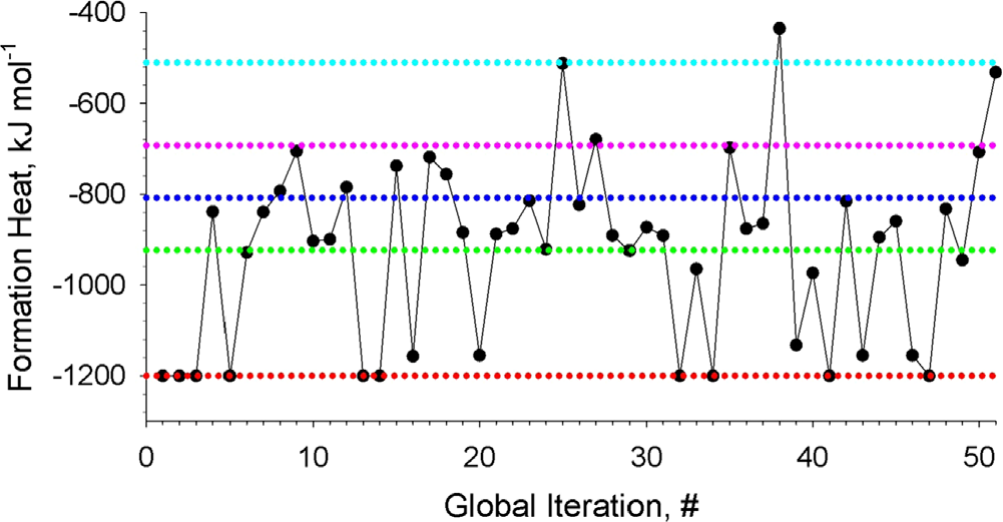
Formation energies of the revealed local-minimum structures of Au_18_Ag_2_. The dotted color lines depict energies of the structures, which were obtained most frequently.

**Fig. 4:**
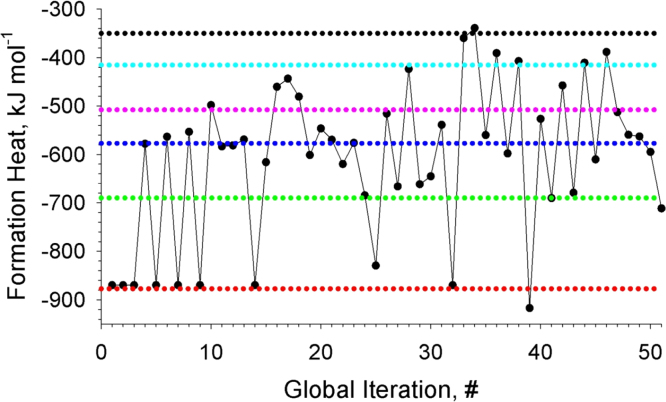
Formation energies of the revealed local-minimum structures of Au_15_Ag_5_. The dotted color lines depict energies of the structures, which were obtained most frequently.

**Fig. 5:**
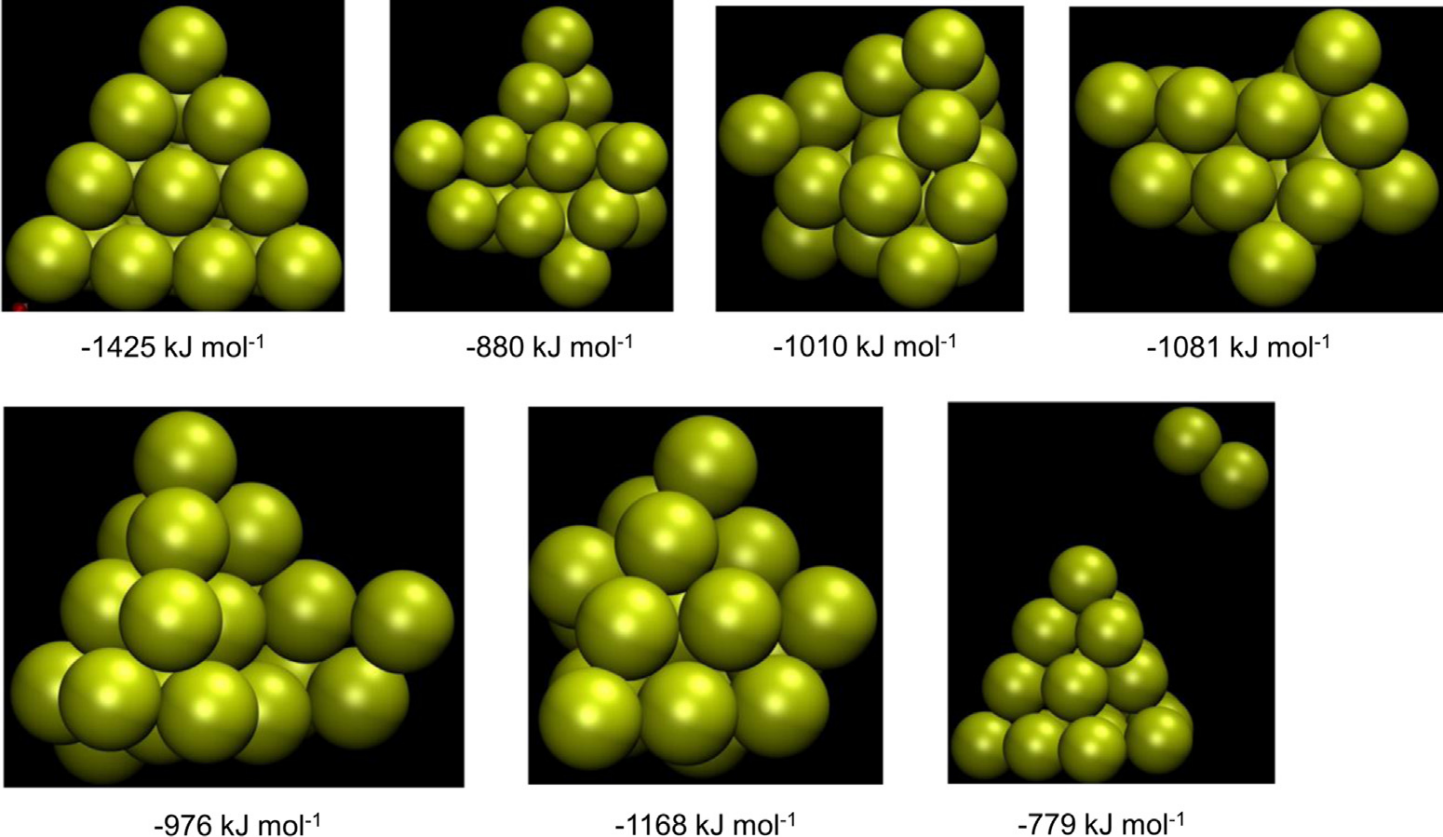
Representative stable structures of Au_20_ and corresponding formation energies.

**Fig. 6:**
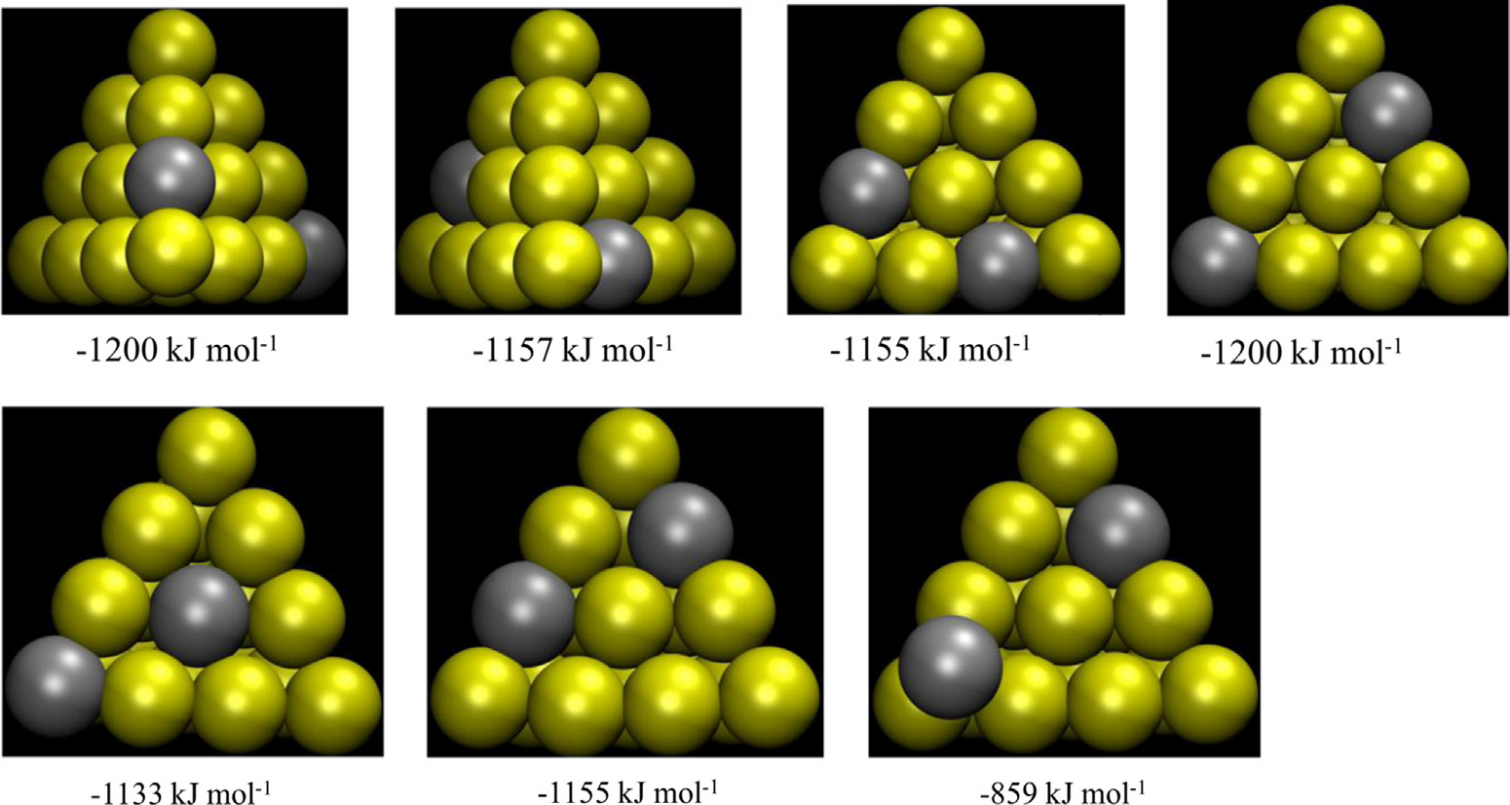
Representative stable structures of Au_18_Ag_2_ and corresponding formation energies.

**Fig. 7:**
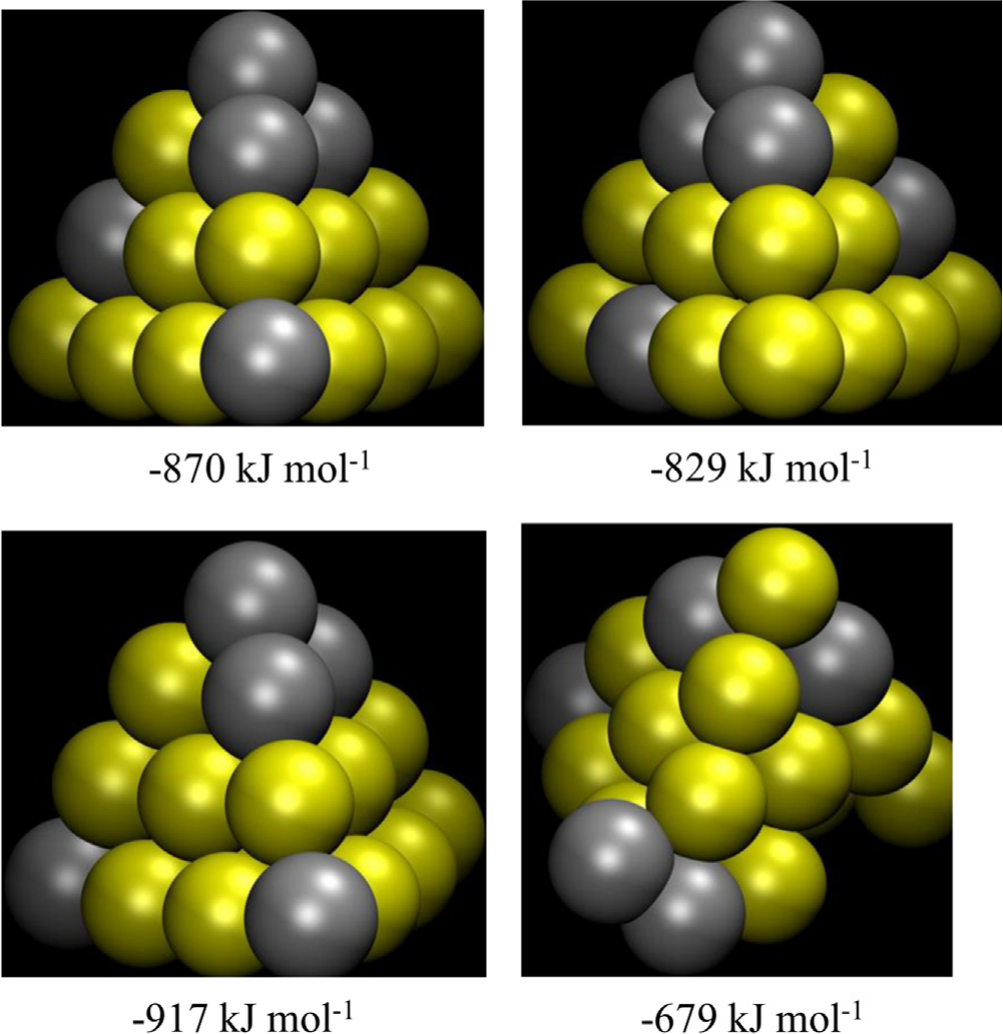
Representative stable structures of Au_15_Ag_5_ and corresponding formation energies.

**Fig. 8:**
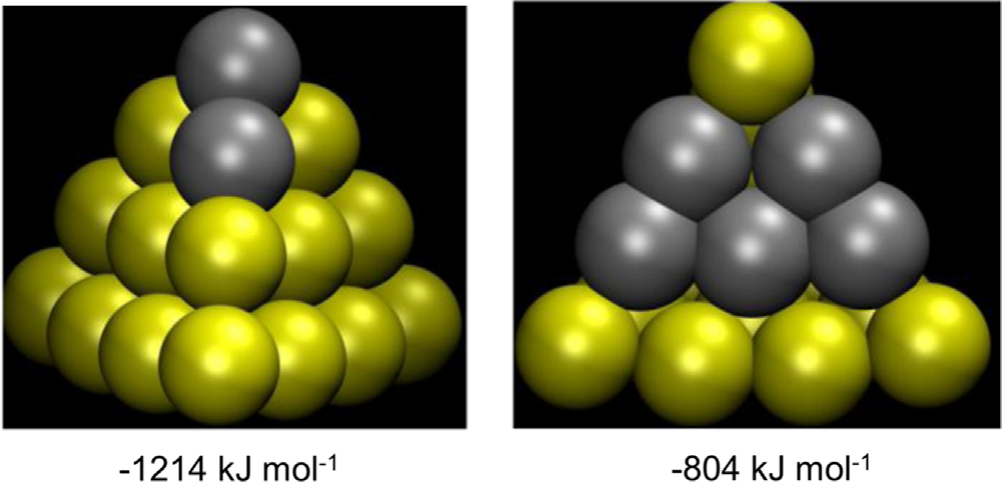
Local-minimum structures and formation energies of Au_18_Ag_2_ and Au_15_Ag_5_ with maximum number of chemical bonds between silver atoms.

The wave functions of the GNCs at every optimization step were represented by means of the PM7 semi-empirical Hamiltonian [2]. PM7 uses the approximation of neglect of diatomic differential overlap, as applied to the Hartree–Fock (HF) method. In turn, all terms of the exact Hamiltonian in HF are expressed as a sum of one-electron operators. Unlike in HF, selected integrals in PM7 are parametrized in view of empirical data and may potentially provide more accurate results than HF. The convergence criterion of the wave function was set to 4.18×10^−4^ kJ mol^−1^(Fig. 1, Fig. 2, Fig. 3, Fig. 4, Fig. 5, Fig. 6, Fig. 7, Fig. 8

The local optimizations were done by means of the Broyden–Fletcher–Goldfarb–Shanno (BFGS) algorithm [3]. This algorithm is essentially failure-proof at the expense of a significant number of iterations (single-point calculations) before it converges. The geometry convergence criterion was set to 1.0 kJ mol^−1^ Å^−1^, which systematically corresponds to less than 0.1 kJ mol^−^^1^ difference in total energy at two consequent BFGS steps (Table 1).

The global optimization was propagated in the framework of the basin hopping (BH) algorithm [4]. Fifty one iterations were performed for every system and the resulting energies were compared to one another. The maximum displacement per gold atom was allowed to be 0.75 Å whereas effective temperature was set to 2000 K. The effective temperature is used to accept or decline possible translations/perturbations within the Metropolis test. For an efficient search, the temperature must be comparable to the higher barrier separating local minima on PES. Since different conformations of GNCs involve different order of bonds between the gold atoms, the bond breakage is required to go from one stable conformation towards another. The effective temperature parameter must be significantly large for the above reason.

The in-home code for navigating PES makes use of the implemented optimization routines provided by SciPy and ASE with minor technical modifications [5]. Implementation of PM7 in MOPAC2012 (openMOPAC.net), as provided by Dr. J.J.P. Stewart, was used. All structures and optimization pathways were visualized in VMD, version 1.9.1 [6]. Input structures were prepared in Gabedit [7].

## Figures and Tables

**Table 1:** Technical details of global minimum search: total number of electrons in each system; number of electrons simulated implicitly; total number of single-point computations (SPCs) performed; the largest number of SPC iterations per one geometry optimization.

Cluster	Total electrons	Implicit electrons	Total number of SPCs	Largest number of SPCs per local optimization
Au_20_	1580	1360	35,350	4611
Au_18_Ag_2_	1516	1296	19,150	742
Au_15_Ag_5_	1420	1200	32,267	3025
